# Drugs, genes and screens: The ethics of preventing and treating spinal muscular atrophy

**DOI:** 10.1111/bioe.12695

**Published:** 2019-11-26

**Authors:** Christopher Gyngell, Zornitza Stark, Julian Savulescu

**Affiliations:** ^1^ Department of Paediatrics University of Melbourne Melbourne Australia; ^2^ Murdoch Children’s Research Institute Melbourne Australia; ^3^ Faculty of Philosophy Oxford Uehiro Centre for Practical Ethics Oxford United Kingdom of Great Britain and Northern Ireland

**Keywords:** disability screening, ethics, pre‐conception screening, spinal muscular atrophy, treatment versus prevention

## Abstract

Spinal muscular atrophy (SMA) is the most common genetic disease that causes infant mortality. Its treatment and prevention represent the paradigmatic example of the ethical dilemmas of 21st‐century medicine. New therapies (nusinersen and AVXS‐101) hold the promise of being able to treat, but not cure, the condition. Alternatively, genomic analysis could identify carriers, and carriers could be offered in vitro fertilization and preimplantation genetic diagnosis. In the future, gene editing could prevent the condition at the embryonic stage. How should these different options be evaluated and compared within a health system? In this paper, we discuss the ethical considerations that bear on the question of how to prioritize the different treatments and preventive options for SMA, at a policy level. We argue that despite the tremendous value of what we call ‘ex‐post’ approaches to treating SMA (such as using pharmacological agents or gene therapy), there is a moral imperative to pursue ‘ex‐ante’ interventions (such as carrier screening in combination with prenatal testing and preimplantation genetic diagnosis, or gene editing) to reduce the incidence of SMA. There are moral reasons relating to autonomy, beneficence and justice to prioritize ex‐ante methods over ex‐post methods.

## 1 INTRODUCTION

Spinal muscular atrophy (SMA) is the most common genetic cause of infant mortality. It affects approximately one in every 12,000 live births1Verhaart, I. E. C., Robertson, A., Wilson, I. J., Asrtsma‐Rus, A., Cameron, S., Jones, C. C., … Lochmüller, H. (2017). Prevalence, incidence and carrier frequency of 5q–linked spinal muscular atrophy – a literature review. *Orphanet Journal of Rare Diseases*, *12*,124. and is caused by a deletion or mutation of the *survival of motor neuron 1* (*SMN1*) gene. The most common form of SMA (SMA1) is inherited in a recessive manner, meaning that someone must inherit two gene mutations to develop the disease, one from each parent. Couples who each have one *SMN1* mutation have a 25% chance of having an affected child. In many cases, the diagnosis comes as a shock, with there being no family history of SMA1.

Babies with SMA1 deteriorate rapidly in infancy, and are often unable to crawl, sit, roll over or turn their heads. Eventually, they lose the ability to swallow and breathe. Life expectancy is less than two years.2Park, H. B., Lee, S. M., Lee, J. S., Park, M. S., Park, K. I., Namgung, R., & Lee, C.(2010). Survival analysis of spinal muscular atrophy type I. *Korean J. Pediatr.*, *53,* 965–970. https://doi.org/10.3345/kjp.2010.53.11.965.


Fortunately, a new wave of interventions is being developed that can reduce the burden of SMA1. The first approved treatment belongs to a class of synthetic drugs called antisense oligonucleotides, which are designed to alter the synthesis of proteins. The drug nusinersen alters the protein produced by the gene *SMN2*, to mimic the absent SMA1 protein. It is delivered into the central nervous system through direct spinal injection. Clinical trials have shown that infants with SMA1 who are administered nusinersen are more likely to achieve major motor milestones (51% of infants treated with nusinersen versus 0% of controls) and were 47% less likely to die than those in the control group.3Finkel, R. S., Mercuri, E., Darras, B. T, Connolly, A. Kuntz, N. Kirschner, J....De Vivo, D. (2017). Nusinersen versus sham control in infantile‐onset spinal muscular atrophy. *New England Journal of Medicine, 377*,1723–1732. It is unclear how long it can extend life for, in those who respond well to the treatment. It is estimated to cost US$750,000 for the initial treatment and then US$375,000 per year from then on.4Institute for Clinical and Economic Review. (2018). *Spinraza® and Zolgensma® for spinal muscular atrophy: Effectiveness and value*. Retrieved from https://icer-review.org/wp-content/uploads/2018/07/ICER_SMA_Draft_Evidence_Report_122018-1.pdf [Accessed 4 September 2019].


Other treatments are being developed for SMA1. A gene therapy treatment called AVXS‐101 has just been given Orphan Drug Designation status by the Food and Drug Administration (FDA) and is currently undergoing clinical trials. AVXS‐101 relies on a virus that has been genetically modified to contain a functional copy of the *SMN1* gene. The modified virus is housed inside a capsid, a protein shell that keeps it isolated and prevents it from replicating. This capsid is introduced into the body through an injection into the bloodstream. It crosses the blood–brain barrier, enters cells, and starts producing the SMN1 protein. AVXS‐101 is less invasive than nusinersen, and is delivered as a one‐off injection. To date, it has only been tested for its safety profile, but it already shows signs of significantly extending survival and promoting the achievement of motor milestones.5Finkel, R. S., Mercuri, E., Darras, B. T, Connolly, A. Kuntz, N. Kirschner, J....De Vivo, Det al., op. cit. It has been given the indicative pricing of US$2.125 million for a one‐off treatment

Both these treatments come at a time when it is becoming cheaper to determine whether individuals carry *SMN1* mutations, and are therefore at risk of having a child with SMA. Traditional carrier screening methods only looked for a small set of mutations associated with SMA.6Prior, T. W. (2008). Carrier screening for spinal muscular atrophy. *Genet. Med.*,*10,* 840–842. The reduced cost of whole genome and whole exome sequencing has led to ‘next generation’ carrier tests, which can identify mutations associated with over 400 diseases simultaneously.7See for example Kirk, E. P., Barlow‐Stewart, K., Selvanathan, A., Josephi‐Taylor, S., Worgan, L., Rajagopalan, S., … Roscioli, T. (2018). Beyond the panel: Preconception screening in consanguineous couples using the TruSight One ‘clinical exome’. *Genetics in Medicine* 21(3), 608–612. https://doi.org/10.1038/s41436-018-0082-9
 Such pre‐conception testing can then be combined with prenatal testing and pregnancy termination, or in vitro fertilization (IVF) and preimplantation genetic diagnosis (PGD) to prevent the birth of children with SMA (as well as other genetic diseases).

Gene editing technologies, such as the CRISPR‐Cas9 system, may provide yet another way to treat SMA in the future. CRISPR‐Cas9 uses an engineered enzyme to make precise modifications to DNA sequences. Laboratory experiments have shown that CRISPR‐Cas9 can correct pathogenetic mutations at the embryonic stage.8Ma, H., Marti‐Gutierrez, N., Park, S.‐W., Wu, J., Lee, Y., Suzuki, K., … Mitalipov, S. (2017). Correction of a pathogenic gene mutation in human embryos. *Nature* ***,*** *548*, 413–419. While germline gene editing has been used reproductively in one case,9Regalado, A. (2019). Rogue Chinese CRISPR scientist cited US report as his green light. *MIT Technology Review*. Retrieved from https://www.technologyreview.com/s/612472/rogue-chinese-crispr-scientist-cited-us-report-as-his-green-light/ [Accessed 02 November 2019]. more research is needed before such techniques can be considered safe enough for a clinical setting. However, in the future, it is plausible that they could be used to correct the mutations that lead to SMA. Such technologies could be used to edit embryos produced through IVF so that they contain a functional copy of the *SMN1* gene, or they could be used pre‐conceptionally to alter sperm or egg cells.

This multitude of treatments and prevention options for SMA raises questions regarding treatment prioritization. When there are multiple therapeutic approaches to SMA available to public health systems, which ought to be funded and supported?10The existence of multiple treatment options also creates questions of prioritization from the perspective on the individual, i.e. if a couple both have a history of SMA in their family, which options for the treatment prevention of SMA should they pursue?


In this paper, we investigate the ethical aspects of treatment prioritization for SMA. To help structure our discussion, we distinguish between ‘ex‐post’ and ‘ex‐ante’ approaches to the treatment and prevention of SMA. Ex‐post interventions are those that are administered after the diagnosis of an infant with SMA (such as nusinersen and AVXS‐101), whereas ex‐ante interventions are those that prevent cases of SMA from occurring in live‐born infants. We argue that individuals and states have moral reasons to pursue both ex‐ante and ex‐post approaches to treating SMA. In some contexts, we should prioritize carrier screening (which enables ex‐post approaches) over treatments like nusinersen. We show that there are moral reasons of autonomy (Section 3), beneficence (Section 4) and justice (Section 5) to prioritize carrier screening. We begin by discussing the disability rights criticism of genetic screening and applying it to the specific case of SMA.

## 2 DISABILITY RIGHTS CRITIQUES AND SMA

A prominent strand of criticism of reproductive technologies that prevent disability comes from the disability rights community.11Asch, A., & Barlevy, D. (2012). Disability and genetics: A disability critique of pre‐natal testing and pre‐implantation genetic diagnosis (PGD). In: eLS. John Wiley & Sons, Ltd: Chichester. DOI: 10.1002/9780470015902.a0005212.pub2. Parens, E., & Asch, A. (1999). Special supplement: The disability rights critique of prenatal genetic testing reflections and recommendations. *Hastings Center Report*, *29*(5), S1–22. Selection against disability is morally problematic, according to these views, for at least two reasons: it expresses morally problematic and hurtful attitudes, and it exemplifies an intolerance to diversity.

The first reason is often discussed under the banner of ‘The Expressivist Argument’.12Edwards, S. D. (2004). Disability, identity and the ‘expressivist objection’. *Journal of Medical Ethics*, *30*(4), 418–420; Hofmann, B. (2017). ‘You are inferior!’ Revisiting the expressivist argument. *Bioethics*, *31*(7), 505–514. The Expressivist Argument claims that when people choose not to implant an embryo, based solely on the fact that it is predisposed to develop a disability, they express an immoral or otherwise objectionable attitude towards existing disabled individuals. Specifically, they express the judgment that the disabled person is *worse* or in some way *less worthy of existence* than other persons. Such judgements are representative of challenges disabled persons face in their daily lives. Through the act of selection, parents make a judgement about an entire embryo (that it is not as preferable as other embryos), solely on the basis of the knowledge that it is predisposed to develop a disability. This mirrors the type of discrimination disabled people are exposed to, where the mere knowledge that a person has a disability is taken to imply facts about the individual as a whole.

In contrast, *treating* a person who is disabled does not express these same problematic attitudes. Treatment does not involve a judgement that an individual as a whole is more or less worthy of existence than others – it may simply represent the belief that an individual would benefit in some way from a particular intervention.

The second line of criticisms from the disability rights community to screening is that it demonstrates an intolerance to diversity. This argument can be pursued in at least two ways. First, it can be argued that the selection against disability shows a negative attitude towards embracing diversity in the family. When parents select against an embryo, based solely on the knowledge that it is predisposed to develop a disability, they demonstrate a problematic attitude towards parenthood, namely that they will only love their child if it has certain traits and characteristics. Good parents should embrace the diverse possibilities that children give rise to. In the words of Michael Sandal, they should be ‘open to the unbidden’13Sandel, M. (2004). The case against perfection. *Atl. Mon.*, *293*, 51–62.. Selection against disability is incompatible with these attitudes.

Furthermore, selection against disability might be symbolic of a problematic attitude to diversity in society. Selection against disability can be taken to imply that society would be better off in the absence of disability. But such views are challenged by at least two considerations. The first is that being disabled can be a source of different perspectives that strengthen society rather than weaken it.14Garland‐Thomson, R. (2012). The case for conserving disability. *J. Bioeth. Inq.*, *9*(3), 339–355. Disability is a source of *narrative* resources, as the lives and struggles of disabled people provide a counterpoint to abled‐body lives and increase our understanding of what it means to be human. Disabilities also provide *epistemic* resources, because the experience of being disabled provides distinctive ‘ways of knowing’ and makes possible new forms of aesthetic expression and evaluation.15Ibid. Relatedly, disability also provides a *cognitive* resource, as the experience of being disabled leads one to develop distinctive perspectives and heuristics.16Gyngell, C., & Douglas, T. (2018). Selecting against disability: The liberal eugenic challenge and the argument from cognitive diversity. *Journal of Applied Philosophy, 35*(2), 319–340. Recent empirical work has shown that cognitive diversity can be more important than individual ability for our collective capacity to solve complex problems.17Page, S. E. (2007). *The difference: How the power of diversity creates better groups, firms, schools, and societies*. Princeton, NJ: Princeton Univ. Press; Gyngell, C., & Easteal, S. (2015). Cognitive diversity and moral enhancement. *Camb. Q. Healthcare Ethics*, *24*(1), 66–74. The presence of disabled people in our population can thus have wide social benefits.18Gyngell & Douglas, op. cit. note16, pp. 319–340.


Secondly, it is argued that many of the negative aspects of disabilities can be seen as socially constructed rather than as being objective features of the condition itself.19Liachowitz, C. H. (1988). *Disability as a social construct :Legislative roots*. Philadelphia: University of Pennsylvania Press. If society were arranged differently, the argument goes, those with disabilities would experience fewer disadvantages associated with their condition, and parents would not be motivated to select against disabilities.

In sum, the disability critique of genetic screening gives us reason to be more sceptical of using PGD than ex‐post approaches. Interestingly, these views can also be interpreted as implying that the use of gene editing is less problematic than the use of genetic selection to avoid disability. Whereas genetic selection expresses a view that some embryos are more worthy of existence than others, gene editing potentially involves modifying a single embryo. It is much more analogous to standard treatment than genetic selection.20Gyngell, C., Douglas, T., & Savulescu, J. (2017). The ethics of germline gene editing. *Journal of Applied Philosophy*, *34*(4), 498–513.


The conceptual basis of the disability critique, as well as its force and scope, have been challenged.21Harris, J. (2001). One principle and three fallacies of disability studies. *Journal of Medical Ethics*, *27*(6), 383–387; Harris, J. (2005). Reproductive liberty, disease and disability. *Reproductive BioMedicine Online*, *10*, 13–16; McMahan, J. (2005). Causing disabled people to exist and causing people to be disabled. *Ethics*, *116*(1), 77–99. Most notably, many argue that selection against disability need not express a negative opinion about disability or disabled people at all.22Nelson, J. L. (1988). The meaning of the act: Reflections on the expressive force of reproductive decision making and policies. *Kennedy Ins.t Ethics J.*, *8*(2), 165–182.  Rather than engaging with these debates here, we will simply note that disability critiques apply less clearly to conditions like SMA1, which cause death in early childhood.

There are no adults with SMA1 who could be directly harmed by the attitudes expressed by testing and selection against SMA1. It might be countered that selection against SMA1 expresses negative attitudes towards those with milder forms of SMA. But here it seems that we can point to the very obvious difference between SMA1 and milder versions: SMA1 is lethal in infancy. Given the obvious disvalue of early death, rejection of a lethal disease does not imply negative attitudes toward milder versions of it. When embryo screening is done with the intention of preventing the severe form of SMA, this need not imply negative assessments of milder versions.

For the same reasons, selection against SMA1 does not symbolise a problematic attitude to diversity. Differences that result in childhood death are certainly not the type of difference we believe that parents should be indifferent to and embrace as part of their ‘openness to the unbidden’. Far from being a source of novel, narrative, epistemic and cognitive resources, SMA1 deprives those affected from developing a unique way of being.

For these reasons, we must look beyond the disability rights critiques of selection to help us decide how to prioritize different interventions that can reduce the disease burden of SMA.

## 3 AUTONOMY

Autonomy is a rich, multifaceted concept that is ubiquitous in philosophy and medicine. The term strictly translates to self‐rule and was originally used by Ancient Greek philosophers to refer to a property of cities.23Stirrat, G. M. (2005). Autonomy in medical ethics after O’Neill. *Journal of Medical Ethics*, *31*(3), 127–130. Greek city‐states were referred to as ‘autonomous’ if they were governed by laws that were of their own making, rather than having laws imposed on them by external powers. Since the 18th century, autonomy has increasingly been applied to individuals rather than collectives. Drawing on the traditional understanding of autonomy, Kant argued that for a person to be autonomous, they had to live in accordance with moral principles that were of their own making, rationally derived and personally endorsed.24Johnson, R., & Cureton, A. (2019). Kant’s moral philosophy. In E.N. Zalta (Ed.), *The Stanford Encyclopedia of Philosophy*. Metaphysics Research Lab., Stanford University. Retrieved from https://plato.stanford.edu/entries/kant-moral/ [Accessed 3 November 2019]. Today, the notion of autonomy is increasingly used in a less formal sense to signify something like the ability of persons to make decisions without outside interference.25Coggon, J., & Miola, J. (2011). Autonomy, liberty, and medical decision‐making. *Camb. Law J.*, *70*(3), 523–547.


Rather than take a stand on how best to understand autonomy, we will instead focus on two values that are promoted by individual autonomy and which can be seen as independently important in medicine: sovereignty and non‐alienation.26Enoch, D. (2017). Hypothetical consent and the value(s) of autonomy. *Ethics,* *128*(1), 6–36. A concern for sovereignty reflects the fact that some decisions are simply the domain of individuals, regardless of how they will exercise this capacity. The idea of sovereignty can be clearly understood through examples that involve property rights. If you own a car, you have sovereignty over it. It is your decision whether a friend can borrow it, no matter how strong your friend’s moral claims might be to borrow the car (e.g. they want to drive someone to the hospital). Decisions about the car are simply yours to make, as you have sovereignty over it. Similarly, the concept of ‘bodily sovereignty’ reflects the idea that people have innate sovereignty over their body and have the ultimate say over decisions that concern it. In reproductive medicine, sovereignty is most commonly associated with the concept of ‘reproductive choice’. Decisions about whether to try to have children, to have sex, to access assisted reproductive technologies, etc. are ultimately the domain of individuals.

Non‐alienation describes the interest of individuals to be the author of their lives and to feel connected with their life’s trajectory. Decisions about weighty medical procedures, with different costs and benefits, should prima facie be left up to the individual to make in accordance with their preferences and values, to ensure a connection with the direction their life is taking.

Considerations of non‐alienation seem natural to apply to decisions about procreation. Procreative decisions have a significant impact on the trajectory of one’s life. If people have a prima facie interest to be the author of their own lives, we ought to give parents wide scope to choose how and when they procreate, which extends to the use of reproductive technologies such as PGD.

The late political philosopher Ronald Dworkin was the first to describe the right to ‘procreative autonomy’, while stressing the importance of leaving weighty decision up to individuals:

The right of procreative autonomy has an important place…in Western political culture more generally. The most important feature of that culture is a belief [in] individual human dignity: that people have the moral right—and the moral responsibility—to confront the most fundamental questions about the meaning and value of their own lives for themselves, answering to their own consciences and convictions….The principle of procreative autonomy, in a broad sense, is embedded in any genuinely democratic culture.27Dworkin, R. (2011). *Life's dominion*. New York: Vintage, pp. 166–167.

The idea of procreative autonomy has been further developed in the work of John Harris28Harris, J. (2000). Clones, genes, and reproductive autonomy. The ethics of human cloning. *913*, 209–217. and John Robertson.29Robertson, J. A. (2003). Procreative liberty in the era of genomics. *Am. J. Law Med., 29*(4), 439–487.


Having a child with a serious disability would significantly affect how one’s life would go. Although raising a child with a disability can be highly rewarding, and give rise to different types of parenting experiences, it is often a very different experience to raising a non‐disabled child. A concern for non‐alienation suggests that people who are having a child should be free to influence whether their child has a disability or not, and make this choice according to their preferences and values.30Such considerations also suggest that parents have an interest in choosing to have a disabled child. We take no stand on this issue in this paper. We note though that other moral considerations discussed below (beneficence and justice) might count against this. Of course any such interests are only prima facie, and in some cases may be defeated by other considerations such as those considered in Section 1 and in Sections 3 and 4 below.

With respect to SMA, considerations of sovereignty and non‐alienation give us reasons to promote all treatment types, be they ex‐post or ex‐ante, as each increases the number of options available to parents in different circumstances. This implies firstly that we have reason to make carrier testing available to couples thinking of having a child. The first major decision facing most parents who are at risk of having children with SMA is whether to conceive through IVF and use PGD, to conceive naturally and undertake prenatal testing and pregnancy termination, or to conceive naturally without genetic testing. The availability of carrier screening thus enables a greater range of reproductive choices for parents. While ex‐post therapies technically give parents a choice, parents are often not in a position to decline effective therapy for their child (and nor would most want to), as in many instances this will fall outside the zone of parental discretion.31Gillam, L. (2016). The zone of parental discretion: An ethical tool for dealing with disagreement between parents and doctors about medical treatment for a child. *Clinical Ethics, 11*, 1–8.


While considerations of autonomy support policies of offering carrier screening, it does not help settle the question of which method of treating or preventing SMA (nusinersen, gene therapy, PGD, gene editing, prenatal testing and termination) is morally superior. The concept of parental autonomy defines the scope of free parental choice in which parents can make decisions free from the interference of others. It does not generate moral reasons in favour of decisions that parents happen to endorse for children. In other words, parental autonomy establishes the right of parents to make decisions on behalf of their children. It does not bear on the question of how parents ought to exercise this right or what the state ought to prioritize. Furthermore, interventions that support autonomy may conflict with other moral considerations. It is therefore essential to consider how other ethical principles bear on SMA prioritization.

## 4 BENEFICENCE

The principle of beneficence is used widely in medicine and philosophy.32Munyaradzi, M. (2012). Critical reflections on the principle of beneficence in biomedicine. *Pan Afr. Med. J.*, *11* (29). As a principle of medical ethics it is often used to denote ‘the doing of good, the active promotion of good, kindness and charity’,33Beauchamp, T. L., & McCullough, L. B. (1984). *Medical ethics: The moral responsibilities of physicians*. Englewood Cliffs, NJ Prentice‐Hall. or the moral obligation ‘to act for the benefit of others, helping them to further their important and legitimate interests, often by preventing or removing possible harms’.34Beauchamp, T. (2011). *The principle of beneficence in applied ethics*. Retrieved from https://stanford.library.sydney.edu.au/archives/fall2011/entries/principle‐beneficence/ [Accessed 28 March 2019].


How we should understand beneficence in a medical context is a complex issue. It partly depends on how one understands the goals of medicine. According to some views, the goals of medicine should be to promote health and to cure disease.35Callahan, D. (1998). Managed care and the goals of medicine. *J. Am. Geriatr. Soc., 46*(3), 385–388. Others argue that medicine should have a broader role, in supporting the overall well‐being of patients. 36Gawande, A. (2014). *Being mortal: Medicine and what matters in the end*. New York, NY: Henry Holt.Complicating matters is that neither health nor well‐being has an accepted definition—and indeed health is sometimes defined as a state of well‐being.37Preamble to the Constitution of the World Health Organization as adopted by the International Health Conference, New York, 19–22 June, 1946; signed on 22 July 1946 by the representatives of 61 states (Official Records of the World Health Organization, no. 2, p. 100) and entered into force on 7 April 1948


It is beyond the scope of this article to delve into any of these issues in any detail. Rather, we will adopt a general view that beneficence is supported by increasing the years of life and quality of life. Both these factors are regularly used as a guide to medical practice.38Jacobs, J. (2009). Quality of life: what does it mean for general practice? *Br. J. Gen. Pract.*, 59(568), 807–808. While ‘quality of life’ is itself a contested term and can be understood in a multitude of ways,39Aksoy, S. (2000). Can the ‘quality of life’ be used as a criterion in health care services? *Bull. Med. Ethics*, *162*, 19–22. these specifics will matter little when considering whether beneficence provides moral reasons to distinguish between different interventions for SMA.

For now, let us put aside the ‘ex‐ante’ preventive options for SMA (PGD, prenatal testing and termination, and gene editing) and focus on ‘ex‐post’ treatments such as nusinersen and AVXS‐101. As nusinersen and AVXS‐101 are so new, we do not have enough information about them to estimate their likely effects on lifespan and quality of life. The only thing we know with certainty is that nusinersen is likely to be more invasive than AVXS‐101. It requires regular intrathecal injections (into the fluid‐filled space of the spinal canal), which can be painful and risk side‐effects, whereas AVXS‐101 is a one‐off intravenous treatment. Until we have more empirical data about these treatments, it is difficult to compare them in terms of beneficence.

Even when we have these additional empirical facts, beneficence may not provide reasons to choose one over the other. Consider two hypothetical treatments, treatment A and treatment B. Treatment A extends lifespan by 10 years but requires regular painful interventions. Treatment B extends life by 8 years but only requires a one‐off painless injection. Which is preferable in terms of beneficence? In other words, is a life of 10 years with regular invasive interventions better than a life of 8 years without such interventions? Such questions are extremely difficult. While empirical studies often try to estimate how much certain conditions (such as blindness, deafness, etc.) reduce one’s quality of life, such comparisons are often radically indeterminate. In such cases, beneficence is not a clear guide about which treatments to prioritize, and we will need to consider other ethical values, or defer to parental autonomy.

How does beneficence apply to ‘ex‐ante’ interventions? Consider PGD first. Imagine a couple, Beth and Jerry, who are both carriers for SMA. They wish to avoid having a child with SMA and conceive through IVF and test each embryo for the presence of the *SMN1* mutations. They implant an embryo, Craig, who does not have the disease.

Who benefits from this intervention? Of course, Beth and Jerry will benefit, as their preference to have a child free from SMA is fulfilled—but does Craig benefit? According to one understanding of benefit, ‘an action benefits a person only if it makes her better off in some respect than she would have been, had the action not been performed’.40Gardner M. (2016). Beneficence and procreation. *Philos. Stud.*, *173,* 321–336. Imagine if Beth and Jerry do not conceive through IVF. Does this leave Craig in a worse off state? No, rather he will not exist at all. Craig himself does not benefit from the action of his parents for there is no scenario where Craig exists and has SMA. In contrast, when we treat a child with nusinersen, or some other ex‐post therapy, we make that child better off than they would be if treatment were withheld. Initially, then, considerations of beneficence seem to favour ex‐post over ex‐ante interventions.

However, there are different ways to understand the requirements of beneficence. We could concede that  PGD does not benefit ‘Craig’, but rather benefits Beth and Jerry’s ‘next child’, whoever that particular person may be.41Parfit, D. (2017). Future people, the non‐identity problem, and person‐affecting principles. *Philosophy & Public Affairs, 45*, 118–157. https://doi.org/10.1111/papa.12088 .
 The relevant comparison to make is between Craig’s well‐being and the well‐being of the child Beth and Jerry would have had, had they not engaged in PGD. In cases where that next child would have had SMA, PGD confers a significant benefit. This way of understanding beneficence denies that there is ‘a counterfactually better‐off condition on benefiting’,42Gardner, M. (2016). Beneficence and procreation. *Philos. Stud., 173*(2), 321–336. so we only benefit others if we make them better off in some sense than they would have otherwise been. As has recently been argued by Molly Gardner, having this view of beneficence allows us to avoid numerous counterintuitive implications about when an action is beneficial or not.43Ibid.


Such views are equivalent to saying that PGD confers an impersonal, rather than a person‐affecting benefit. That we should understand beneficence in such an impersonal way has long been argued in bioethics. Savulescu’s principle *of procreative beneficence* (PB) states that ‘*couples (or single reproducers) should select the child, of the possible children they could have, who is expected to have the best life, or at least as good a life as the others, based on the relevant, available information*’.44Savulescu, J. (2001). Procreative beneficence: Why we should select the best children. *Bioethics, 15*, 413–426. This appeals to the idea that parents have obligations to benefit their child in general, regardless of their identity.45This view is challenged by others. For example Bennett, R. (2009). The fallacy of the Principle of Procreative Beneficence. *Bioethics*, *23*(5), 265–273.


If we understand beneficence in this broad, impersonal way, then it is clear that beneficence strongly favours PGD over other ex‐post treatments like nusinersen. When PGD is used to avoid SMA, this commonly results in the existence of someone with an average expected level of well‐being. Ex‐post treatments, like nusinersen and AVXS‐101, are unlikely to restore normal life expectancy and motor function, and hence they will be unlikely to match the level of benefit provided by PGD.

What about prenatal testing and termination? As no child is directly produced through termination, at one level no one benefits. Prenatal testing and termination may, therefore, be the least beneficial treatment. However, this neglects the fact that many parents who engage in prenatal testing and terminations will likely try to have another child without SMA and have this explicit motivation when they choose this option. Each subsequent child the couple has will have a 25% risk of having SMA1. However, in cases where termination leads to another child who is SMA‐free, it is, arguably, the equivalent to PGD being used to prevent SMA.

This suggests that in cases where couples are likely to be able to conceive more children, prenatal testing and termination is more beneficent than ex‐post treatments like nusinersen. However, in cases where the couple is unlikely to be able to have another child, it becomes the least beneficial option.

In the future, gene editing may provide a way of completely curing SMA at the embryonic stage. Again, imagine Beth and Jerry, both of whom carry an *SMN1* mutation. They wish to avoid having a child with SMA and conceive through IVF. This time, they produce a single embryo, Karen, who has two *SMN1* mutations. Gene editing is used to correct these mutations. Karen has a normal life expectancy and no disability. Gene editing is thus like PGD, in that it results in a baby without SMA1 and, all else being equal, a normal life expectancy. Hence it is a highly beneficial option.46One reason why gene editing may be different from PGD is that it could bestow these benefits in an identity‐preserving way. There is a possible scenario in which Karen exists, and has SMA (that scenario where her parents do not use gene editing technologies on the embryo). Hence gene editing may result in person‐affecting rather than impersonal benefits, depending on one’s view of personal identity. This would be relevant for those who believe that person‐affecting benefits are more important than impersonal harms. Of course, while gene editing has risks of off‐target mutations, genetic selection is in one way safer and preferable.47Savulescu, J., Hemsley, M., Newson, A., & Foddy, B. (2006). Behavioural genetics: Why eugenic selection is preferable to enhancement. *J. Appl. Philos., 23*,157–171.


## 5 JUSTICE

The Roman lawyer Cicero gives one of the earliest definitions of justice as ‘the virtue which assigns to each his due’.48Cicero. (1933). *On the nature of the Gods. Academics. *Translated by H. Rackham. Loeb Classical Library 268. Cambridge, MA: Harvard University Press, p321. This broad definition still captures the core concerns of justice today. While justice encompasses many elements of ethics and law—such as the punishment of crimes, the distribution of resources, and the relationship between society and individuals, it fundamentally represents a concern for giving people what they are ‘due’. In moral philosophy, justice is seen as a particularly powerful value. If something is labelled as ‘unjust’—be it an institution, policy, or individual action—this generates strong, if not decisive, reason to reject it.49Miller, D. (2017). Justice. In E. N. Zalta (Ed.), *The Stanford Encyclopedia of Philosophy* (Fall 2017 Edition). Retrieved from https://plato.stanford.edu/archives/fall2017/entries/justice/ [Accessed 5 April 2019].


We can distinguish between different domains of justice, including distributive justice (how to distribute resources justly), corrective justice (how to compensate people for past wrongdoing done to them) and procedural justice (how people are treated to establish rules and regulations).

In medicine, questions of justice usually focus on distributive justice. Medical resources, such as drugs, specialist time, pathology tests etc., are limited. The demand for these resources outstrips their availability. This raises the question of how to allocate them in a way that ‘assigns each their due’.

How should we distribute limited medical resources, such as access to SMA treatments? Such questions have mattered very little until the last few decades. Historically there were few treatments for most rare diseases, and certainly not multiple effective treatments for a single catastrophic condition. However, advances in medicine and technology mean that states must now make decisions about which treatments to develop and fund, and therefore which goals they should pursue when distributing medical resources.

As we saw in Section 3, a simple metric that can be used to compare alternative treatments is their effect on survival. One view, then, is that a just distribution of medical resources would be the distribution that improves the life expectancy of a population the most. However, as we saw in Section 3, there are problems with metrics that focus merely on survival. People care about more than just surviving: they care about what they can do with that survival. Any theory of the just distribution of medical resources must take this into account.

To accommodate the fact that the *quality* of one’s life matters in addition to its *duration*, economist Alan Williams proposed the measure of the quality‐adjusted life year (QALY) in 1985. This measure takes one year of life, in good health,50Good health is measured through surveys of public attitudes towards different health states. to be worth 1, and a year of life in a suboptimal state to be worth some value less than 1, depending on how badly quality of life is affected.

QALYs have become a standard measure that healthcare systems use to distribute medical resources. Many different health states have been assigned QALY values, based on empirical attitudinal studies looking at their effect on well‐being. For example, being blind for a year is estimated to be the equivalent of 0.61 QALYs,51Schwander, B. (2014). Early health economic evaluation of the future potential of next generation artificial vision systems for treating blindness in Germany. *Health Economics Review, 4*, 27. https://doi.org/10.1186/s13561-014-0027-1. meaning that healthcare systems should prioritize treatments that prolong lifespan for two years in good health (and generate two QALYs) over those that prolong life for three years but result in blindness (and generate 1.83 QALYs).  While, such empirical work is highly controversial, these difficulties do not affect our subsequent analysis.52For example see Pettitt, D.A., Raza, S., Naughton, B., Roscoe, A., Ramakrishnan, A., Ali, A….. Brindley, D.A. (2016). The limitations of QALY: A literature review. *Journal of Stem Cell Research and Therapy, 6*(4), pp. Article: 1000334.


Increases in QALYs essentially track increases in quality and years, which we discussed in Section 3. As we saw, ex‐ante interventions that prevent SMA can be expected to generate more well‐being than ex‐post interventions that treat it after the diagnosis. Accordingly, they also generate more QALYs.

However, when considering justice and not beneficence, we must look beyond the amount of QALYs that an intervention generates, to its cost‐effectiveness. Cost determines over a population with limited resources how many people can be treated.When we approach issues through the lens of justice, considerations of the cost of treatments are not merely practical considerations, but also key ethical issues. In public health systems, there is a limited amount of resources. If one individual is treated with an expensive treatment when they could be treated with a cheaper treatment, that reduces the resources available to others. This indirectly harms others who could use this resource. There is an ethical imperative to use public health resources in a cost‐effective way. The cost of treatment is, therefore, an ethical consideration.

As discussed in the Introduction, nusinersen is expected to be very expensive: US$750,000 for the initial treatment, and US$375,000 for each year of subsequent treatments. Let us suppose that the public health system fully funds it, and it manages to extend a patient’s life for 10 years. This means that the course of treatment will cost the health system US$4.125 million. In contrast, PGD will cost approximately US$15,000. If a couple uses PGD rather than nusinersen, this will save over US$4 million of health resources that could be used to help others. Furthermore, PGD generates many more QALYs, as we argued in Section 3. Favouring PGD over nusinersen thereby ensures that more people get access to medical resources, which in turn promotes justice.

The cost of prenatal testing and termination is even cheaper than PGD, with a total cost of approximately US$5000. When this is likely to result in another child, it also generates more QALYs and results in an even more just distribution of resources (assuming the child is SMA‐free).

While the cost of gene editing is unclear at the stage, it will likely be in the range of PGD and IVF, perhaps around US$30,000.

### 5.1 Screening

The above analysis of the cost‐effectiveness of PGD and prenatal testing overlooks a central issue. In our discussions, we have been assuming that couples know that they are carriers for SMA, and thus prenatal testing and PGD are visible options to them. However, this will not be the case for the vast majority of SMA carriers. Only people who have had a DNA carrier test will know with certainty whether or not they carry an *SMN1* mutation, and this will mostly only be if they have a family history and pay for and access carrier screening tests.

What would the cost‐effectiveness of PGD and prenatal testing be if these procedures were accompanied by population‐wide carrier screening? Earlier studies of the cost‐effectiveness of SMA screening found it to be very expensive. One study that looked at the cost‐effectiveness of universal prenatal screening for SMA found that the cost was US$4.9 million per QALY gained.53Little, S. E., Janakiraman, V., Kaimal, A., Musci, T., Ecker, J., & Caughey, A. B. (2010). The cost‐effectiveness of prenatal screening for spinal muscular atrophy. *American Journal of Obstetrics and Gynecology, 202,* 253.e1–253.e7. It should be noted that the study looked at the cost effectiveness of universal ‘prenatal’ screening, rather than at ‘carrier’ screening. However, the costs of carrier screening would be comparable. However, this study has been criticized for looking only at QALY gain by the mother of an affected child,54Grosse, S. D. (2010). What is the value for money of prenatal carrier screening for spinal muscular atrophy? Too soon to say. *American Journal of Obstetrics & Gynecology*, *202*, 209–211. and thus overlooking the fact that screening also generates QALYs through the existence of SMA‐free children who would not exist without the screening programs.

Furthermore, the last few years have seen a vast improvement in the cost‐effectiveness and breadth of so‐called ‘next generation’ screening methods, which can detect carrier status for up to 400 conditions in one test. A study looking at the cost‐effectiveness of population carrier screening for 176 conditions (including SMA) estimated that such programs would cost ~$50,000 per QALY gained.55Beauchamp, K. A., Taber, K. A. J., & Muzzey, D. (2019). Clinical impact and cost‐effectiveness of a 176‐condition expanded carrier screen. *Genet. Med., 21*, 1948–1957.


The relative advantages of population carrier screening combined with PGD are particularly clear when we consider the fact that nusinersen and AXVS‐101 are most effective when administered in pre‐symptomatic infants.56Bertini, E., Hwu, P., Reyna, S., Farwell, W., & De, D. (2016). Phase 2 study design of antisense oligonucleotide nusinersen in presymptomatic infants with spinal muscular atrophy. *Neuromuscular Disorders*, *26*, S210. https://doi.org/10.1016/j.nmd.2016.06.450. To maximize the efficiency of these treatments, they will need to be combined with newborn screening, to allow infants with SMA to be identified and treated quickly.57Boardman, F. K., Sadler, C., & Young, P. J. (2017). Newborn genetic screening for spinal muscular atrophy in the UK: The views of the general population. *Mol. Genet. Genomic Med., 6*, 99–108. https://doi.org/10.1002/mgg3.353. In July 2018, The U.S. Department of Health and Human Services Secretary recommended the inclusion of SMA in the national newborn screening panel, in part to facilitate affected infants being able to access these new therapies.58Miller, N. S. (2018). Spinal Muscular Atrophy added to national newborn screening panel. OrlandoSentinel.com. Retrieved from http://www.orlandosentinel.com/health/os‐sma‐newborn‐screening‐approved‐20180709‐story.html [Accessed Jul 11, 2018]. Newborn screening would add a substantial additional cost, making these treatments even less cost‐effective.

In sum, we have reasons of justice to ensure that medical resources are equitably distributed. This requires that we consider the cost‐effectiveness of potential medical treatments. When people know that they are SMA carriers, there are strong reasons for justice to promote ex‐ante intervention like PGD and prenatal testing and termination, over ex‐post methods like nusinersen. While more empirical work needs to be done to assess the cost‐effectiveness of carrier screening, especially for SMA when this is combined with screening for other Mendelian disorders, it appears highly likely that such programs should also be promoted on justice grounds. There are also strong distributive justice arguments to make carrier testing, PGD and IVF, or prenatal testing and selective termination free of charge to affected couples.

## 6 CONCLUSIONS

A plethora of new interventions promise to reduce the burden of SMA for families. However, they raise difficult questions regarding resource prioritization. In this paper we have argued that the moral principles of autonomy, beneficence and justice support reducing SMA through ex‐ante methods, like PGD, prenatal testing and termination.

A couple using PGD to create a healthy child confers a great benefit to their next child, when their next child would otherwise have had SMA. This is far greater than the expected benefit generated from ex‐post treatments, which are unlikely to cure SMA completely. When couples engage in prenatal testing and termination and are likely to have another child, this is also a highly beneficial option. In cases where termination is unlikely to lead to another child, such approaches are not supported by considerations of beneficence.

There are also reasons of justice to promote ex‐ante methods. PGD and prenatal testing and termination are many times cheaper than ex‐post treatments for SMA. By prioritizing these more cost‐effective therapies, we ensure that medical resources are more equitably distributed.

The advantages of ex‐ante interventions over ex‐post methods suggest that we should also be prioritizing carrier screening for SMA. It is only when prospective parents know that they are SMA carriers that they can make fully informed decisions about which treatments to employ. There are therefore reasons of autonomy to promote carrier screening. Furthermore, insofar as carrier screening will make prenatal testing and PGD more visible options for couples at risk of having children with SMA, such programs will also promote beneficence and autonomy.

This is not to say that this is the only way the moral principles could be applied in this case, or that there are no other moral considerations that could bear on these issues. But, in the absence of counter‐arguments, we believe that we have provided a strong ethical argument in support of prioritizing ex‐ante over ex‐post methods to reduce the burden of SMA.

## CONFLICT OF INTEREST

The authors declare no conflict of interest.

